# Myosins FaMyo2B and Famyo2 Affect Asexual and Sexual Development, Reduces Pathogenicity, and FaMyo2B Acts Jointly with the Myosin Passenger Protein FaSmy1 to Affect Resistance to Phenamacril in *Fusarium asiaticum*

**DOI:** 10.1371/journal.pone.0154058

**Published:** 2016-04-21

**Authors:** Zhitian Zheng, Xiumei Liu, Bin Li, Yiqiang Cai, Yuanye Zhu, Mingguo Zhou

**Affiliations:** College of Plant Protection, Nanjing Agricultural University, Key Laboratory of Pesticide, Jiangsu Province, Nanjing, 210095, China; Seoul National University, REPUBLIC OF KOREA

## Abstract

We previously reported that mutations occurred in the gene myosin5 were responsible for resistance to the fungicide phenamacril in *Fusarium graminearum*. Here, we determined whether there is a functional link between phenamacril resistance and the myosin proteins FaMyo2B and Famyo2 in *Fusarium asiaticum*, which is the major causal agent of Fusarium head blight in China. We found that *FaMyo2B* acts jointly with *FaSmy1* to affect resistance to phenamacril in *F*. *asiaticum*. We also found that *FaMyo2B* disruption mutant and *Famyo2* deletion mutant were defective in hyphal branching, conidiation, and sexual reproduction. Δ*Famyo2* also had an enhanced sensitivity to cell wall damaging agents and an abnormal distribution of septa and nuclei. In addition, the *FaMyo2B* and *Famyo2* mutants had reduced pathogenicity on wheat coleoptiles and flowering wheat heads. Taken together, these results reveal that *FaMyo2B* and *Famyo2* are required for several *F*. *asiaticum* developmental processes and activities, which help us better understand the resistance mechanism and find the most effective approach to control FHB.

## Introduction

*Fusarium graminearum sensu lato* (teleomorph *Gibberella zeae* (Schwein.) Petch) is the main pathogens of Fusarium head blight (FHB) of wheat and other small cereal grains, which can produce harmful mycotoxins in infected grain and threat animals and human [1–4]. So far, altogether 11 phylogenetic species were the causal agent of FHB disease and these species belong to the *Fusarium graminearum* Schwabe species complex of B-trichothecene toxin producers [5–9]. In China, since 1936 when FHB was first reported, FHB disease have become more and more severe in the middle and lower reaches of the Yangtze River, southern winter wheat region, and the northeastern spring wheat region [10]. Zhang et al. [11] reported that 77.3% of the 299 isolates collected from different places experiencing FHB epidemics in China were identified as *F*. *asiaticum* and another 68 isolates were confirmed to be *F*. *graminearum*. So *F*.*asiaticum* is the major pathogen of FHB epidemics in China.

Owing to few crop cultivars with natural resistance to *Fusarium* are available, the control of FHB has been depended on application of fungicides during wheat anthesis in the past few decades [10, 12, 13]. The use of a novel cyanoacrylate compound, phenamacril, reduced both the mycotoxin level and FHB index by 80% [14–17]. Phenamacril-resistant mutants have been readily obtained *in vitro* by fungicide domestication and ultra-violet (UV) irradiation. Most of the resistant mutants were moderately to highly resistant. We recently reported that mutations occurred in the gene myosin5 were responsible for resistance to phenamacril in *F*. *graminearum* [18] and Zhang et al. [19] found that JS399-19 (phenamacril) inhibited ATPase activity of FgMyo1 (myosin5) motor domain and JS399-19 (phenamacril) had a serious impact on localization of the wild-type FgMyo1 at the tips of germlings. In *F*. *graminearum*, there are three types of myosins: class II myosin myo2 (FGSG_08719.1) [20], class V myosin myosin2B (FGSG_07469.1), and class I myosin myosin-5 (FGSG_01410.1). In this paper, we used gene heterokaryotic disruption and gene deletion to determine whether the myosin proteins myosin-2B and myo2 could affect resistance to phenamacril in *F*. *asiaticum*.

Myosins are ATPase-dependent molecular motors that are responsible for an interaction with actin filaments. Generally, all of the myosins have a highly conserved ~80-kDa motor domain which generate chemo-mechanical, unidirectional force. So far, 31 myosin family classes have been discovered due to phylogenetic analyses and genomic survey [21]. Class I myosins myosins-I are common actin-dependent motor proteins. Besides the actin-activated ATPase that translocates actin filaments, the myosins-I can trigger Arp2/3 complex dependent actin polymerization by a Tail Homology 2 (TH2) domain, which binds to microfilamentous actin [22]. In addition, The yeast myosin-I Myo5, and its homologue Myo3, were involved in the formation of vesicles at the plasma membrane [23]. Class V myosins are processive motor proteins that transport their cargo toward the barbed (+) ends of actin filaments and they participate in multiple membrane trafficking events [24, 25]. *Saccharomyces cerevisiae* has two class V myosins, the nonessential Myo4 and the essential Myo2. While Myo4 mediates the movement of ER tubules and the transport of mRNAs, Myo2 plays a significant role in the segregation of membrane-bound organelles including peroxisomes, vacuoles, and other organelles of the secretory pathway and in the transport of secretory vesicles [26–29]. The class II myosin Myo1 is either nonessential or essential for viability. However, Myo1 is important for cell wall maintenance in yeast cells [30]. In *S*. *cerevisiae*, the myosin passenger-protein Smy1 transported by myosin V is involved in a negative feedback mechanism which prevents overgrowth and detects cable length [31]. The coiled-coil interaction (CCI) network reveals that Myo1 and Myo2 are interacting proteins that regulate Smy1p; when overexpressed, Smy1p can partially restore for defects, in the Myo2 mutant, compensating polarized growth and overcoming lethality at a restrictive temperature [32–35].

In *F*. *graminearum*, Song et al. [20] identified myo2, a class II myosin gene; they further demonstrated that myo2 is required for septation, conidiation, and sexual reproduction, and is important for pathogenesis and mycotoxin production. In this paper, we found that the class V myosin gene, *FaMyo2B*, in *F*. *asiaticum* affects asexual and sexual development, reduces virulence, and acts jointly with the myosin passenger protein gene *FaSmy1* to affect resistance to phenamacril. Our data suggest that *FaMyo2B* and *Famyo2* help us better understand the resistance mechanism and find an effective approach for FHB control.

## Materials and Methods

### Growth, conidiation, and perithecial assays of strains and mutants

The strains and mutants used in this paper are listed in Table 1. The wild-type phenamacril-sensitive strain 2021 and phenamacril-resistant strain Y2021A with induced phenamacril resistance were used for transformation. For analysis of growth phenotype and growth rate, all the strains were grown at 25°C on PDA (200 g of potato, 20 g of glucose, 15 g of agar, and 1 L of water) for 3 days. To assay the ability of the mutants in response to the cell wall damaging agents, mycelial growth was measured after incubation at 25°C for 3–8 d on PDA plates containing 5 mM caffein and 0.05% (w/v) Congo red. The percentage of mycelial growth inhibition (RGI) was calculated using the formula RGI = ((A-B)/(A-5)) *100, where A is the colony diameter of the control, and B is that of a treatment.

**Table 1:** *Fusarium asiaticum* strains used in this study.

Strain	Genotype	Reference
**2021**	Wild type	[15]
**D2021*FaMyo2B-93***	*FaMyo2B* disruption mutant in 2021 genetic background	This study
**Δ2021*Famyo2-7***	*Famyo2* deletion mutant in 2021 genetic background	This study
**Δ2021*FaSmy1-19***	*FaSmy1* deletion mutant in 2021 genetic background	This study
**Y2021A**	Isolate resistant to phenamacril; generated from the wild-type strain 2021 by fungicide treatment	[36]
**DY2021A*FgMyo2B-3***	*FaMyo2B* disruption mutant in Y2021A genetic background	This study
**DY2021A*FgMyo2B-11***	*FaMyo2B* disruption mutant in Y2021A genetic background	This study
**DY2021A*FgMyo2B-12***	*FaMyo2B* disruption mutant in Y2021A genetic background	This study
**DY2021A*FgMyo2B-3C***	*FaMyo2B* complement mutant in Y2021A genetic background	This study
**DY2021A*FgMyo2B-11C***	*FaMyo2B* complement mutant in Y2021A genetic background	This study
**DY2021A*FgMyo2B-12C***	*FaMyo2B* complement mutant in Y2021A genetic background	This study
**ΔY2021A*Myo2-8***	*Famyo2* deletion mutant in Y2021A genetic background	This study
**ΔY2021A*Myo2-8C***	*Famyo2* complement mutant in Y2021A genetic background	This study
**ΔY2021A*FaSmy1-14***	*FaSmy1* deletion mutant in Y2021A genetic background	This study
**ΔY2021A*FaSmy1-15***	*FaSmy1* deletion mutant in Y2021A genetic background	This study
**ΔY2021A*FaSmy1-40***	*FaSmy1* deletion mutant in Y2021A genetic background	This study

For sporulation production assays, 10 fresh mycelial plugs taken from the periphery of a 3-day-old colony of each strain and mutant were added to a 200-ml flask containing 100 ml of MBL medium (MBL; 30 g of mung beans were boiled in 1 L of water for 15 min, and the mixture was then filtered through cheesecloth). Each strain and mutant was represented by three flasks. The flasks were incubated at 25°C for 7 days with shaking (185 rpm). The number of conidia in the MBL medium in each flask was measured with a hemacytometer and microscope. Sexual reproduction on carrot agar plates were assayed as previously described [37]. All the experiments were performed three times.

### Construction of vectors for the disruption and deletion mutants and complementation using double-joint PCR technique and transformation

The construction of gene heterokaryotic disruption and gene deletion vectors of *F*. *asiaticum* were carried out using the methods as previously described [17]. The primers used to amplify the flanking sequences for each gene are listed in Supporting Information S1 Table. Putative gene heterokaryotic disruption and gene deletion mutants were identified by PCR assays with primers (Supporting Information S1 Table) and Southern blot assays.

One of the *Famyo2* deletion mutants (Δ*Y2021AFamyo2*-8) was complemented with the full length *Famyo2* gene to confirm that the phenotypic changes of the *Famyo2* deletion mutant were due to the deletion of the gene. The vector for the complementation of *Famyo2* was amplified from the genomic DNA of strain Y2021A using primers P9/P12 (S1 Table). Before this vector was transformed into strain Y2021A, *Famyo2* in the vector was sequenced to ensure the flawlessness of the sequence. The complemented strain was designated Δ*Y2021AFamyo2*-8C.

To complement *FaMyo2B* heterokaryotic disruption mutants, The *FaMyo2B* complement plasmid pNEO-*FaMyo2B*-Com was constructed using the plasmid pNEO [38]. The full-length *FaMyo2B* gene, including the 1757-bp upstream and 150-bp terminator regions, was amplified from genomic DNA of strain Y2021A with primer P15/P16 (S1 Table), and cloned into the XbaI-SbfI site of pNEO to generate the complement plasmid pNEO-FaMyo2B-Com. Transformation of D*Y2021AFgMyo2B-3*, D*Y2021AFgMyo2B-11* and D*Y2021AFgMyo2B-12* with the complement plasmid pNEO-FaMyo2B-Com as well as other deletion and disruption vectors were conducted as described previously [17], except that neomycin (100 mg/mL) was used as a selection agent.

### Microscopic examination of mycelial and ascospores

For investigation of the morphology of conidia, ascospores, and hyphae, freshly harvested conidia, 2-week-old perithecia, and fresh mycelial taken from colonies of each strain and mutant that had been grown on a thin layer of water agar for 36 h was examined with an Olympus IX-71 microscope (Tokyo, Japan). Young hyphae that grew from conidia in YEPD liquid medium (w/v, 1% peptone, 0.3% yeast extract, 2% glucose) for 12 h were collected for microscopy by staining with 5 μg ml^-1^ DAPI and 10 μg ml^-1^ CFW (both from Sigma) for 5 min. Images were taken from two independent experiments.

### Sensitivity to phenamacril

Sensitivity to phenamacril (experiment code JS399-19), which was provided by the Jiangsu Pesticide Institute Co. and had been recognized by International Fungicide Resistance Action Committee (FRAC) in 2015, was assessed for all the strains and mutants listed in Table 2. Mycelial plugs (5 mm in diameter) taken from the periphery of a 3-day-old colony were placed on the centre of PDA plates amended with phenamacril at: 0, 0.025, 0.05, 0.1, 0.2, or 0.4 μg/mL for sensitive strains; 5, 10, 25, 50, or 100 μg/mL for strains with intermediate sensitivity; or 25, 50, 100, 200, or 400 μg/mL for resistant strains. Three replicates for each concentration were used for each strain and mutant. After cultures were incubated at 25°C for 3 to 8 days, colony diameters were measured; the diameter (5 mm) of the original mycelial plugs were subtracted from each measurement. the 50% effective concentration (EC_50_) values of strains and mutants were calculated by regressing percentage growth inhibition against the log of fungicide concentration with DPS software. The experiment was performed twice.

**Table 2:** Phenotypes of *Fusarium asiaticum* wild-type strain 2021, resistant strain Y2021A, and mutants in terms of growth, conidiation, and pathogenicity.^a^

Strain	Growth rate^b^ (cm/day)	Conidia produced (×10^5^/ml)	Percentage of diseased spikelets (%)^c^	Lesion length on stem (cm)^d^
**2021**	2.55±0.02A	44.75±6.72A	94.55±1.20A	2.61±0.28A
**D2021*FaMyo2B-93***	2.31±0.05B	3.47±1.03C	17.59±6.68B	0.52±0.19B
**Δ2021*Famyo2-7***	1.36±0.08D	0.25±0.07D	2.84±0.40C	0.06±0.07C
**Y2021A**	2.58±0.03A	42.00±8.49A	96.58±1.30A	2.78±0.26A
**DY2021A*FaMyo2B-3***	2.21±0.05C	6.15±1.48D	19.56±5.87B	0.43±0.16B
**DY2021A*FaMyo2B-3C***	2.52±0.04A	31.62±6.24B	NA	NA
**ΔY2021A*Famyo2-8***	1.43±0.01D	0.18±0.04C	2.92±0.18C	0.06±0.08C
**ΔY2021A*Famyo2-8C***	2.49±0.06A	40.05±5.37A	NA	NA

^**a**^Values are means and standard deviations. Means in a column followed by the same letter are not significantly different (P = 0.01).

^**b**^Growth rate and conidiation were measured after incubation of three replicates for 3 and 7 days, respectively. Growth rate was measured on PDA plates. Conidiation was measured in flasks containing MLB.

^**c**^Percentage of diseased spikelets per spike 14 days after inoculation. Thirty spikes were inoculated for each strain.

^**d**^The length of brown lesions on diseased stems 14 days post inoculation. Ten coleoptiles were inoculated for each strain. NA, not analysis.

### Plant infection and pathogenicity

Flowering wheat heads of cultivar Zhenmai 5 (which is sensitive to *F*. *asiaticum* and is widely cultured in China) were inoculated with 10 μl of conidia suspensions (1.0 ×10^5^ conidia ml^-1^) as previously described [39]. Thirty replicate wheat heads were inoculated for each strain and mutant. After inoculation, each wheat head was covered with a plastic bag for 2 days to maintain moisture, and scab symptoms were examined at 15 d post-inoculation (dpi).

For wheat coleoptiles assays, 3-day-old seedlings of wheat cultivar Zhenmai 5 were used according to previous method with modifications [40]. 3 days after seeds were sown, the top 2 to 3 mm of the coleoptiles were cut, and the tip was injected 10μl conidial suspension. For each strain and mutant, 10 coleoptiles were inoculated and maintained in the growth chamber at 25°C and with 95% relative humidity. The experiment was repeated twice. The pathogenicity of the strains and mutants were assessed by measuring the length of brown lesions on diseased stems at 14 dpi.

### Quantitative RT-PCR (qRT-PCR)

RNA was extracted with the RNAsimple kit (Tiangen) from germ-tubes grown for 18 h in YEPD liquid medium. The cDNAs were amplified with the PrimeScript^®^ RT reagent kit (TaKaRa). Quantitative PCR experiments were performed using an ABI 7500 real-time detection system (Applied Biosystems, USA) with an SYBR Green reaction mix containing 10 μL of 2 × SYBR green premix, 2 μL of template, 0.4 μL of forward primer (10 mM) and reverse primer (10 mM), and 7.2 mL of nucleotide-free water. The PCR program included an initial denaturation step at 95°C for 30 s and then 40 amplification cycles at 95.0°C for 5 s and 60°C for 34 s. To ensure specificity, only primers that generated a single peak in the melting curve were selected. Primers used for qRT-PCR analysis are listed in S1 Table and glyceraldehyde-3-phosphate dehydrogenase (GAPDH) was used as a reference. Each experiment included two technology replicates and was repeated at least three times.

### Yeast two-hybrid analysis

The coding sequence of each tested gene was amplified from the cDNA of *F*. *asiaticum* strain 2021 with primer pairs for constructing yeast two-hybrid plasmids. The cDNA fragment of *FaSmy1* encoding 933 amino acids was inserted into the yeast GAL4-binding domain vector pGBKT7 and the cDNA fragment of *FaMyo2B* and *Famyo2* encoding the tail domain (790 amino acids and 743 amino acids, respectively) were inserted into GAL4 activation domain vector pGADT7. The pairs of yeast two-hybrid plasmids were transformed into *S*. *cerevisiae* strain AH109 together following the LiAc/SS-DNA/PEG (lithium acetate/single-stranded DNA/ polyethylene glycol) transformation protocol [41]. The plasmid pair pGADT7 and pGBKT7-53 served as a positive control, and the plasmid pair pGADT7 and pGBKT7-Lam served as a negative control. Transformants were cultured at 30°C for 3 days on synthetic medium (SD) lacking Trp and Leu and then were transferred to SD lacking of Leu, His, and Trp. Three independent experiments were conducted to confirm the yeast two-hybrid assay results.

## Results

### Sequence analysis and characterization of *FaMyo2B* and *Famyo2* in *F*. *asiaticum*

We identified the *F*. *asiaticum* other myosin genes *FaMyo2B* (FGSG_07469) and *Famyo2* (FGSG_08719) by a BLASTP search of the Fusarium genome (http://www.broadinstitute.org/annotation/genome/fusarium_group/MultiHome.html) using the *S*. *cerevisiae* Myo1 and Myo2 proteins as queries. FaMyo2B (GenBank accession no. ESU13735.1) was predicted to encode a 1583 amino acid protein which shares 46% similarity with *S*. *cerevisiae* class V myosin Myo2. Famyo2 (GenBank accession no. ESU14588.1) was predicted to encode a 2342 amino acid protein which shares 24% similarity with *S*. *cerevisiae* class II myosin Myo1.

The DNA sequences of an *FaMyo2B* locus (ranging from 234 bp upstream to 150 bp downstream of the *FaMyo2B* coding region) and an *Famyo2* locus (ranging from 281 bp upstream to 318 bp downstream of the *Famyo2* coding region) from strains 2021 and Y2021A of *F*. *asiaticum* were retrieved by PCR amplification using primers 07469F/07469R and 08719F/08719R (S1 Table). *FaMyo2B* has an open reading frame (ORF) of 5,095 bp and six introns, and *Famyo2* has an ORF of 7,300 bp and four introns. In addition, multiple sequence alignment of *FaMyo2B* and *Famyo2* revealed that their motor domains are conserved to those of other myosin orthologues (S1–S3 Figs). This region comprises the myosin superfamily domain.

### Disruption of *FaMyo2B* and deletion of *Famyo2* and complementation of both of the mutants

For a detailed functional analysis of *FaMyo2B* and *Famyo2*, we generated deletion mutants or heterokaryotic disruption mutants by transformation of the gene replacement cassette *HPH-HSV-tk* in a phenamacril-sensitive strain (2021) and phenamacril-resistant strain (Y2021A) of *F*. *asiaticum* (Table 1). Putative deletion strains were determined by PCR amplification using different primer pairs and southern blot analysis using genomic DNA of the parental strain and mutants (S4A–S4D Fig). When we targeted *FaMyo2B* for gene deletion, we recovered 217 *HPH-*resistant and *HSV*-sensitive transformants, but all were ectopic mutants, perhaps because *FaMyo2B* encodes an essential gene in *F*. *asiaticum* as it does in *S*. *cerevisiae* citation. In spite of the highly efficient homologous integration events in *F*. *asiaticum*, the failure to obtain an *FaMyo2B* null mutant strongly revealed a lethal impact of *FaMyo2B* deletion in this fungi. However, when we identified these transformants by PCR and southern blot, we found that some transformants indicated gene replacement cassette *HPH-HSV-tk* integration at the left junction and right junction but the results of southern blot indicated two bands, one is wild-type band and another is deletion mutant band. So we thought we got *FaMyo2B* heterokaryotic disruption mutants and we used these mutants for further functional study.

To complement the *Famyo2* deletion mutants, One of the Δ*Famyo2* mutants (Δ*Y2021AFamyo2*-8) was complemented with the parental gene *Famyo2*. The putative complementations were examined by PCR and southern blot analysis (S4B and S4D Fig). And to complement the *FaMyo2B* disruption mutants, D*Y2021AFgMyo2B-3*, D*Y2021AFgMyo2B-11* and D*Y2021AFgMyo2B-12* were complemented with the complement plasmid pNEO-*FaMyo2B*-Com. The putative complementation were selected by neomycin and examined by southern blot analysis (S4D Fig).

### *FaMyo2B* and *Famyo2* are involved in regulating hyphal growth and conidiation

**Fig 1:**
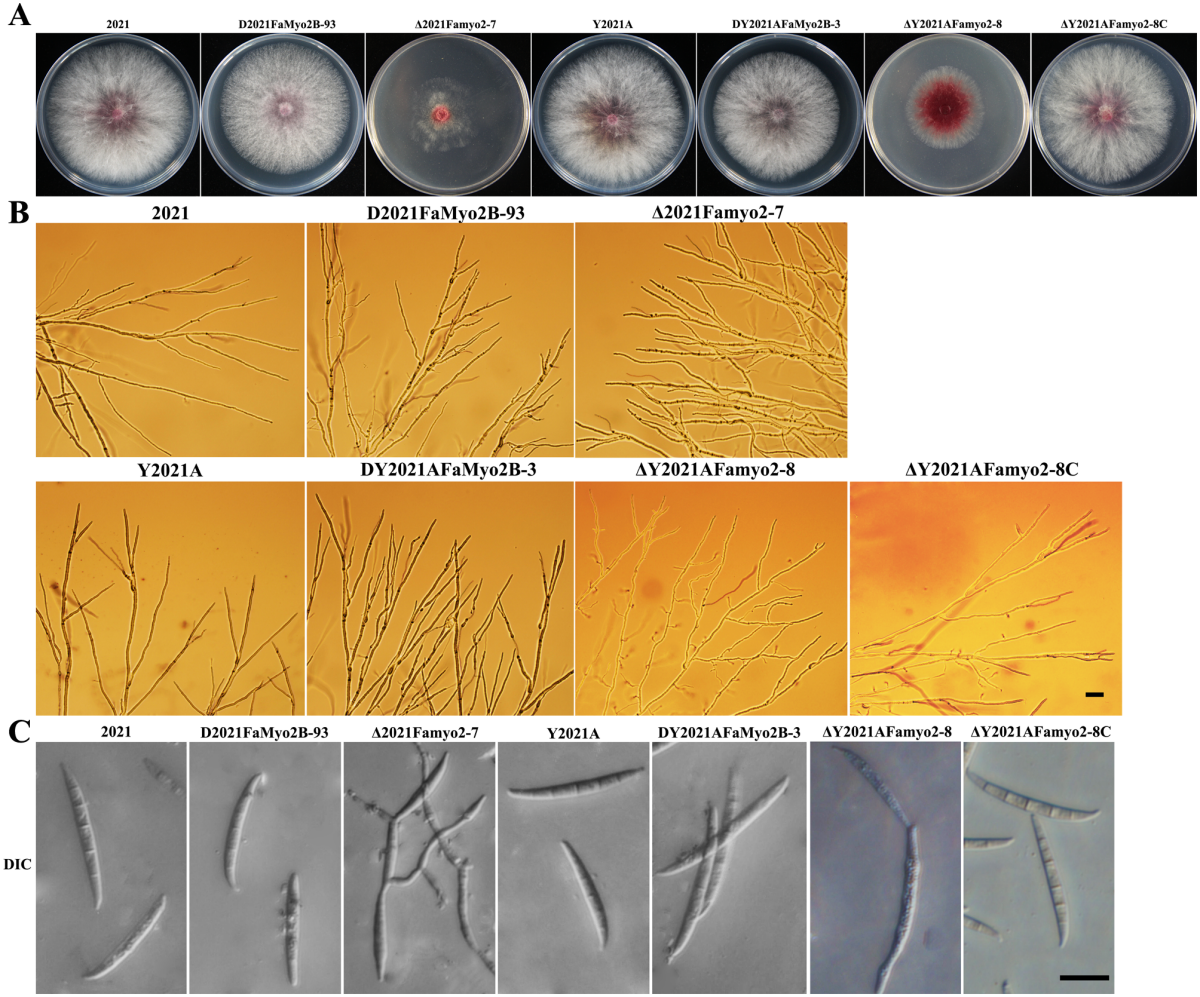
Colony and conidia morphology and hyphal tip growth and branching patterns of the wild-type 2021, the *FaMyo2B* disruption mutant, the *Famyo2* deletion mutant and the *Famyo2* complement mutant. (A) Colonies were photographed after 3 days at 25°C on PDA. (B) The branching angles of the hyphae were reduced and distorted in the extension zone of the mutant colonies that had grown on a thin layer of water agar for 36 h. Bar = 200 μm. (C) Photographs of conidia obtained in the conidiation assay (see text). Bar = 20 μm.

As shown in Fig 1, mycelial growth on potato dextrose agar (PDA) was slightly reduced by disruption of *FaMyo2B* but greatly reduced by deletion of *Famyo2*. Δ*Famyo2* mutant grew prominently slower than the progenitor strains 2021 and Y2021A on PDA plates and had a distinctive colony morphology but the complementation of *Famyo2* deletion mutants restored the morphology (Fig 1A). Microscopic examination indicated that the hyphae of the *FaMyo2B* heterokaryotic disruption mutant generally branched at a narrower angle than the parental strains (Fig 1B), and that the hyphae of the *Famyo2* deletion mutant were distorted, suggesting that *FaMyo2B* and *Famyo2* play a major role in vegetative development.

To assay the effects of *FaMyo2B* and *Famyo2* on conidiation, the strains were incubated in MBL medium with shaking. After 7 days, the *FaMyo2B* and *Famyo2* mutants had produced significantly fewer conidia than the progenitor strains or the complemented strains (Table 2). Moreover, the deletion mutant formed conidia in chains whereas the parental strain formed separate, canoe-shaped conidia (Fig 1C). Disruption of *FaMyo2B* did not significantly affect conidial morphology (Fig 1C).

### Combined effects of FaMyo2B and the myosin passenger protein FaSmy1 on the phenamacril resistance in *F*. *asiaticum*

To determine whether *FaMyo2B* and *Famyo2* affect the level of phenamacril resistance in *F*. *asiaticum*, we conducted fungicide resistance and sensitivity tests. As indicated in Table 3, disruption of *FaMyo2B* significantly reduced phenamacril EC_50_ values for the phenamacril-resistant strain Y2021A but not for the phenamacril-sensitive strain 2021. In contrast, deletion of *Famyo2* did not affect phenamacril resistance for the resistant strain or for the wild-type sensitive strain. Interestingly, the complemented strains partially restored the resistance of the *FaMyo2B* disruption mutants to phenamacril (Table 3).

**Table 3:** Sensitivity of *Fusarium asiaticum* strains to phenamacril.^a^

Strain	EC_50_ (μg/ml)
**2021**	0.19
**D2021*FaMyo2B-93***	0.20
**Δ2021*Famyo2-7***	0.16
**Δ2021*FaSmy1-19***	0.21
**Y2021A**	168.61
**DY2021A*FaMyo2B-3***	21.99
**DY2021A*FaMyo2B-11***	24.88
**DY2021A*FaMyo2B-12***	21.00
**DY2021A*FgMyo2B-3C***	92.61
**DY2021A*FgMyo2B-11C***	95.15
**DY2021A*FgMyo2B-12C***	98.24
**ΔY2021A*Famyo2-8***	161.16
**ΔY2021A*Myo2-8C***	166.30
**ΔY2021A*FaSmy1-14***	52.81
**ΔY2021A*FaSmy1-15***	62.32
**ΔY2021A*FaSmy1-40***	73.49

^**a**^Values are means of three experiments (differences among the experiments were not significant, i.e., P > 0.05, Fisher’s LSD test).

**Fig 2:**
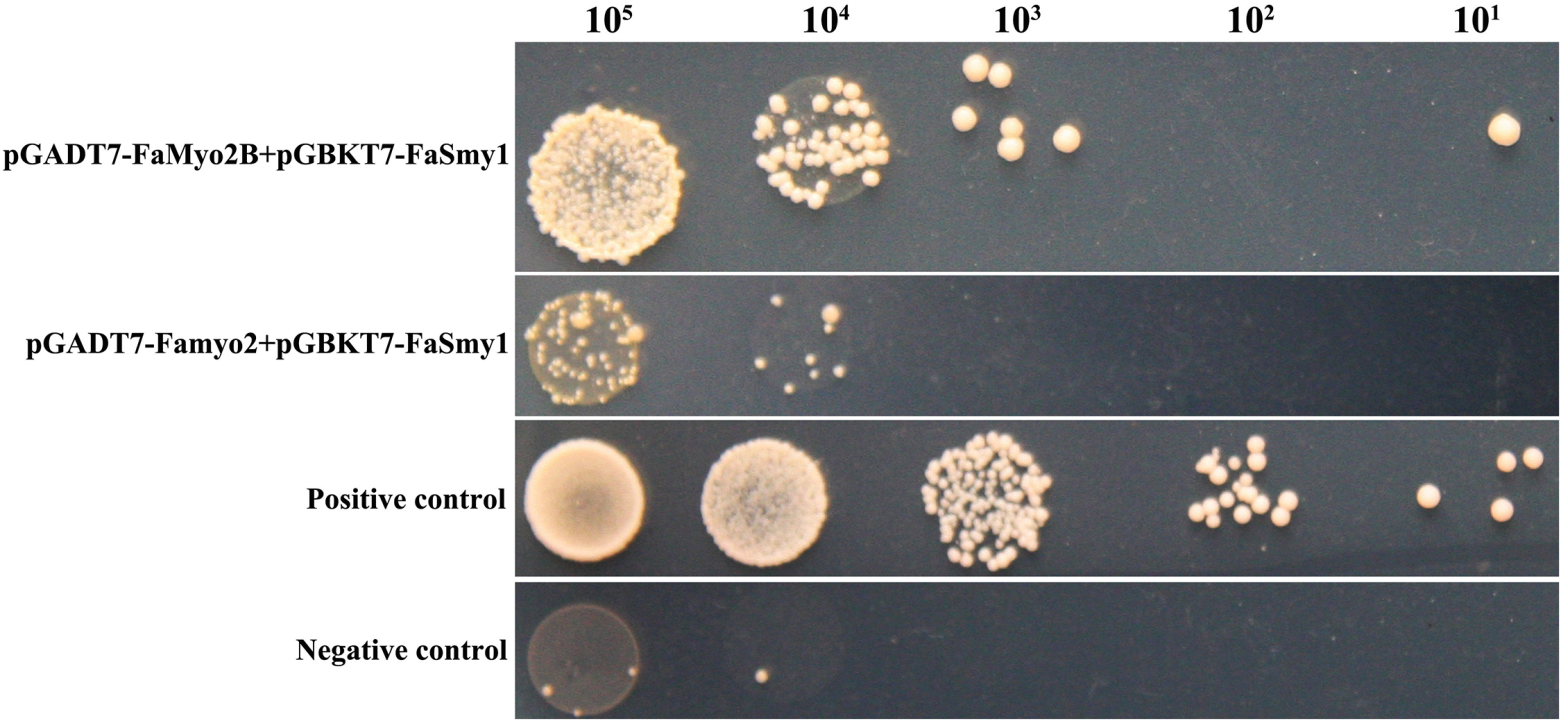
Yeast two hybrid analyses of interactions between FaSmy1 and FaMyo2B and between FaSmy1 and Famyo2 of *Fusarium asiaticum*. Serial dilutions of yeast cells (cells ml^-1^) that were transferred with the prey and bait constructs shown in the figure were determined for growth on yeast minimal synthetic defined base (SD) lacking tryptophan, leucine and histidine. The plasmid pairs pGADT7 and GBKT7-53 was used as a positive control, and the plasmid pairs pGADT7 and pGBKT7-Lam and was used as a negative control.

As the myosin passenger protein, Smy1p physically interacts with the class V myosin Myo2p in budding yeast. In this paper, yeast two-hybrid analysis revealed that FaSmy1 interacts with the tail portion of the FaMyo2B but not with Famyo2 (Fig 2). Furthermore, knockout of *FaSmy1* significantly reduced phenamacril EC_50_ values in the phenamacril-resistant strain Y2021A but not in the sensitive strain (2021A) (Table 3).

**Fig 3:**
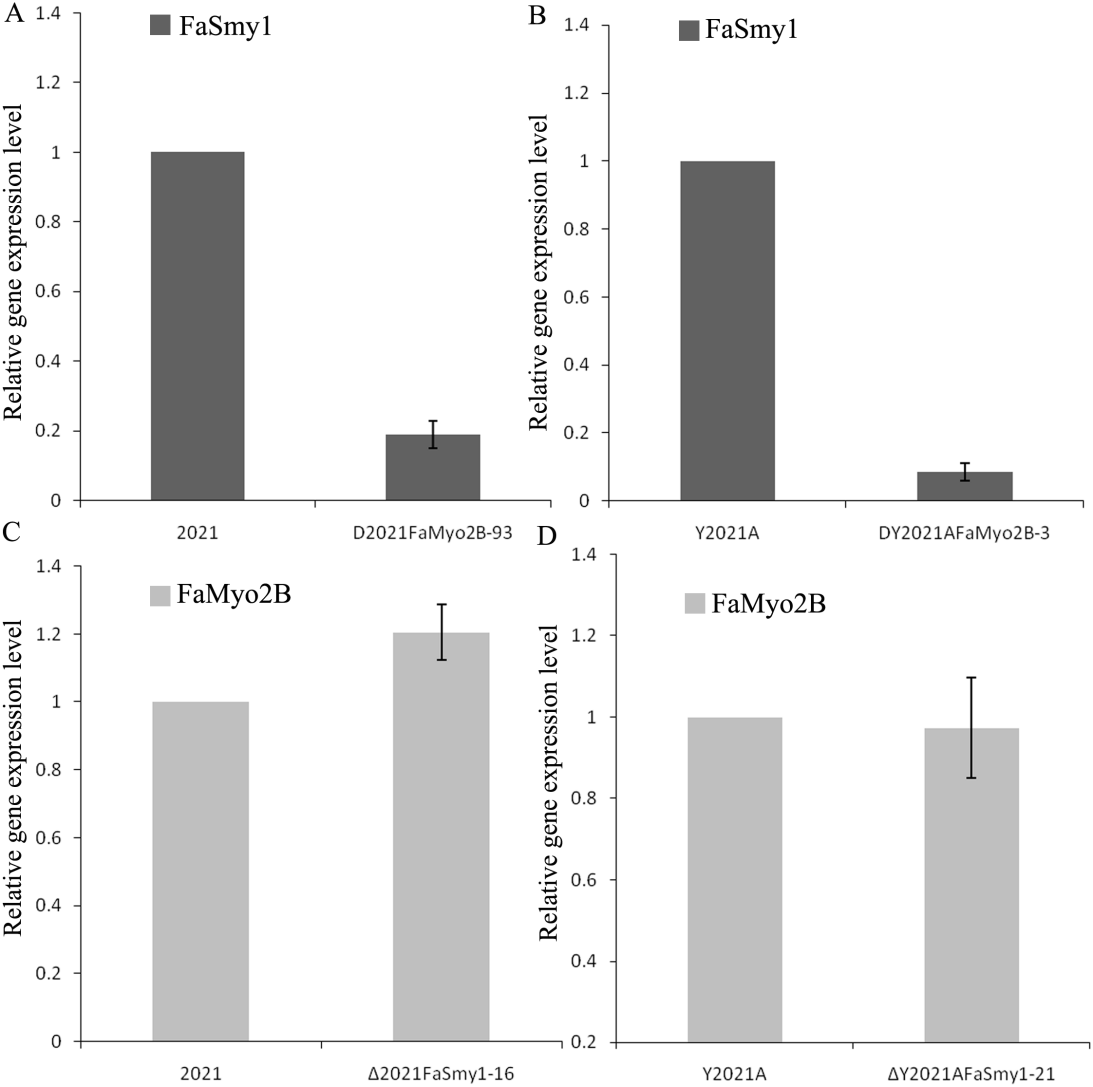
Expression levels of *FaSmy1* and *FaMyo2B* in phenamacril-sensitive strain 2021, phenamacril-resistance strain Y2021A and mutants. Expression levels of *FaSmy1* in the phenamacril-sensitive strain 2021 and in the corresponding *FaMyo2B* mutant (A), and in the phenamacril-resistant strain Y2021A and in the corresponding *FaMyo2B* mutant (B). Expression levels of *FaMyo2B* in the phenamacril-sensitive strain 2021 and in the corresponding *FaSmy1* mutant (C), and in the phenamacril-resistant strain Y2021A and in the corresponding *FaSmy1* mutant (D).Values are the means ± standard error (SE) of three repeated experiments.

**Fig 4:**
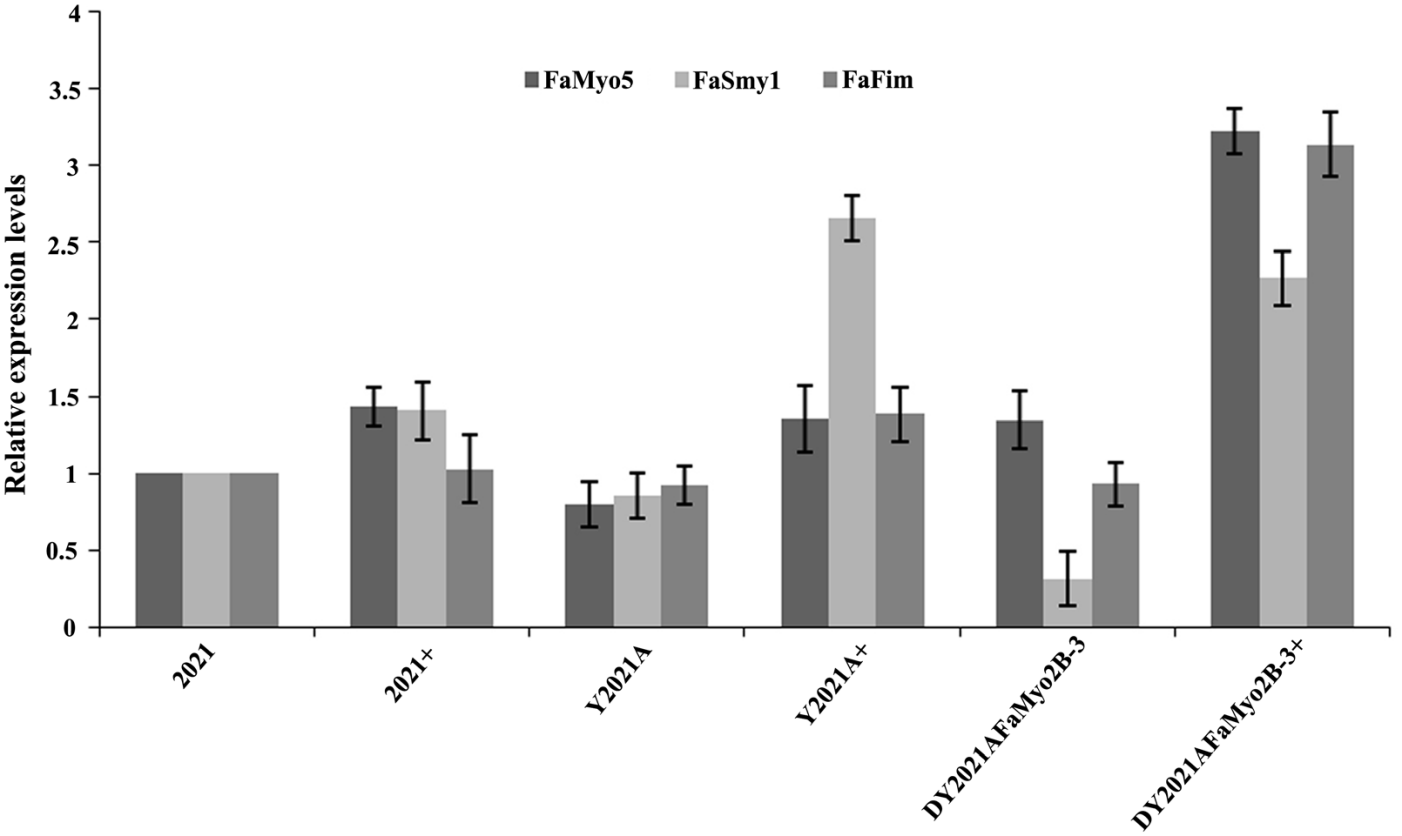
Expression level of *FaMyo5*, *FaFim*, and *FaSmy1* genes in the phenamacril-sensitive strain 2021, the phenamacril-resistant strain Y2021A, and the phenamacril-resistant *FaMyo2B* disruption mutant DY2021A*FaMyo2B-3*. + represents strains treated with phenamacril at 1 μg/ml for 6 h. Values are the means ± standard error (SE) of three repeated experiments.

In *S*. *cerevisiae*, overexpression of Smy1p not only complements Myo2p localization in the deletion mutant, but it also increases the localization of wild-type Myo2p [37]. However, disruption of *FaMyo2B* significantly down-regulated the expression of *FaSmy1* in the phenamacril-sensitive strain 2021 and in phenamacril-resistant strain Y2021A of *F*. *asiaticum* but deletion of *FaSmy1* didn’t affect the expression of *FaMyo2B* (Fig 3). Our previous studies showed that FgFim is a key protein regulating phenamacril resistance and that mutations occurred in the gene myosin5 confer resistance to phenamacril in *F*. *graminearum* [17, 18]. When treated with 1 μg/ml phenamacril for 6 h, the *FaMyo2B* disruption mutant of the phenamacril-resistant strain Y2021A (D*Y2021AFaMyo2B-3*) significantly up-regulated *FaMyo5*, *FaFim*, and *FaSmy1* genes relative to the sensitive strain 2021. However, the phenamacril-resistant strain Y2021A significantly up-regulated only the *FaSmy1* gene (Fig 4). Furthermore, the D*Y2021AFaMyo2B-3* mutant significantly up-regulated the resistance gene *FaMyo5* relative to the resistant strain Y2021A. These results suggest that FaMyo2B acts jointly with the myosin passenger protein FaSmy1 to affect phenamacril resistance in *F*. *asiaticum*.

### *FaMyo2B* and *Famyo2* are essential for sexual reproduction

**Fig 5:**
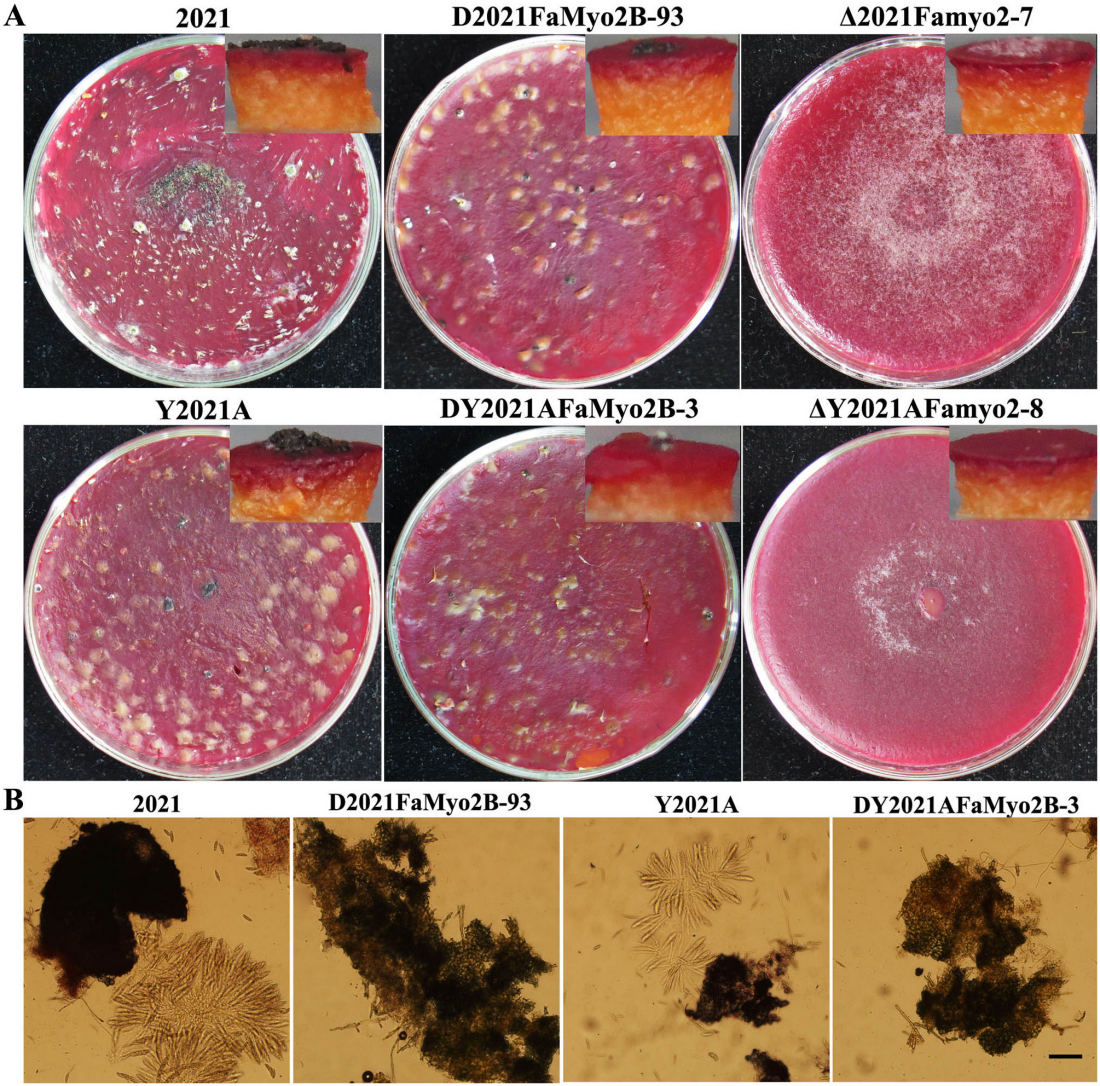
Effects of *FaMyo2B* disruption and *Famyo2* deletion on the sexual development of *Fusarium asiaticum*. (A) Cultures were photographed after 2 weeks of growth on carrot agar. The insets show that the relative numbers of perithecia produced by each strain. (B) Morphology of ascospores in 2-week-old perithecia. Bar = 80 μm.

Because *F*. *graminearum* is a homothallic fungus, sexual reproduction plays an important role in its infection cycle [42, 43]. Previous study has shown that Δ*myo2* mutant strains of *F*. *graminearum* produced no perithecia when cultured on wheat kernels for 14 days [20]. Δ*Famyo2* mutants of *F*. *asiaticum* also failed to form perithecia on carrot agar plates (Fig 5A), which showed that the mutant may be defective in female fertility. However, the parental strains produced obvious perithecia and discharged ascospores on carrot agar plates. Interestingly, the *FaMyo2B* mutant produced normal perithecia but did not produce viable ascospores in 2-week-old perithecia (Fig 5B). Thus, we conclude that *FaMyo2B* and *Famyo2* are essential for sexual reproduction.

### *FaMyo2B* and *Famyo2* affect pathogenicity of *F*. *asiaticum*

**Fig 6:**
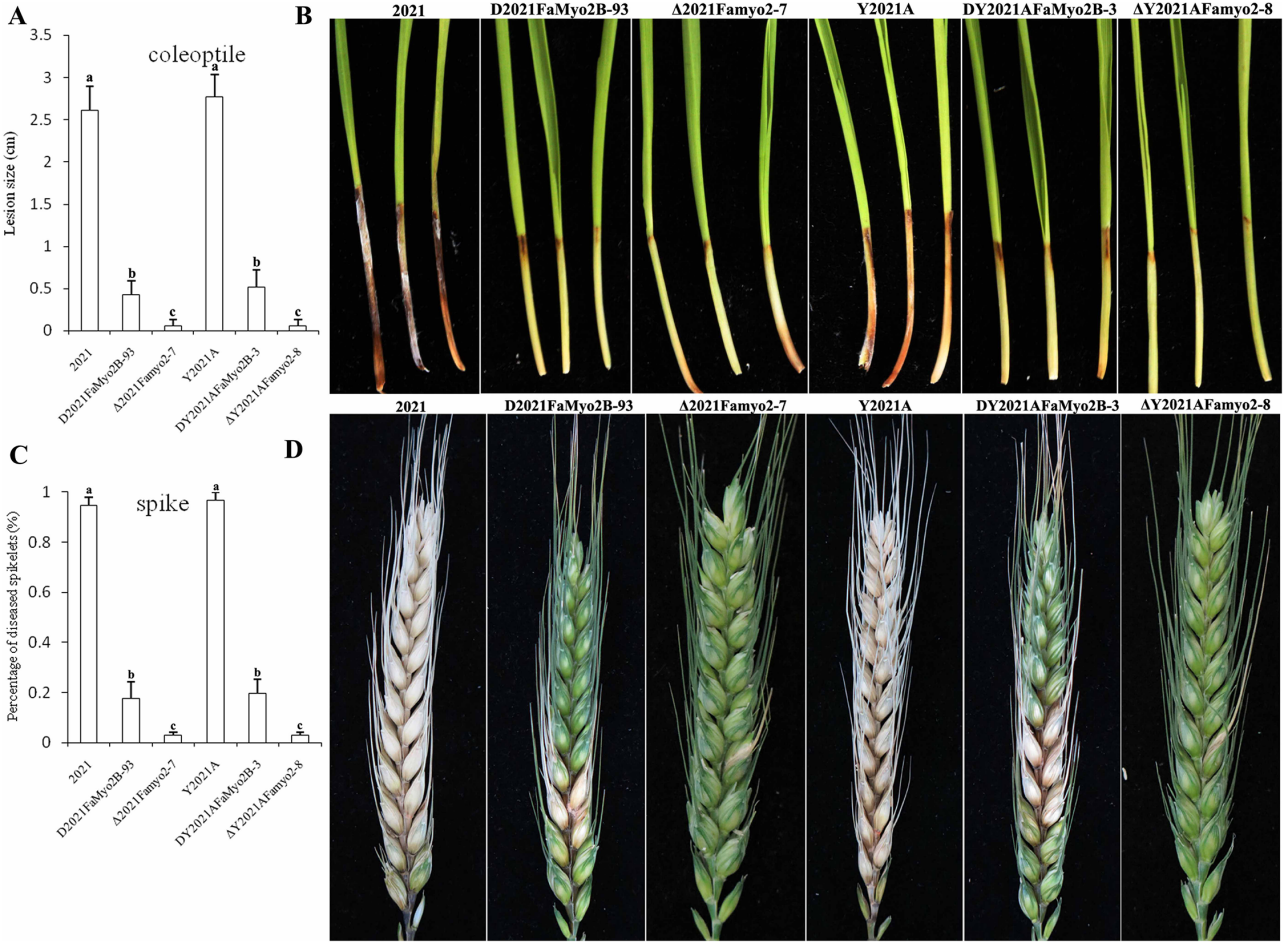
Virulence of *FaMyo2B* and *Famyo2* mutants. (A) Three-day-old seedlings were inoculated with conidial suspensions of the wild-type 2021, the *FaMyo2B* mutant, and the *Famyo2* mutant. The lengths of brown lesions of wheat coleoptiles were measured 14 days post inoculation. (B) Infected wheat coleoptiles were photographed 15 days after inoculation. (C) Percentage of diseased spikes on inoculated wheat heads. (D) Infected wheat heads were photographed 14 days after inoculation.

In infection assays with flowering wheat heads, the *FaMyo2B* disruption mutants caused obvious scab symptoms on the inoculated wheat kernels and were able to infect nearby spikelets (Table 2), but their virulence was reduced by ~80% relative to the virulence of the parental strains (Fig 6C and Table 2). In contrast, the two Δ*Famyo2* mutants caused nearly no disease symptoms on most of the spikelets inoculated with Δ*2021Famyo2-7* or Δ*Y2021AFamyo2-8* even 15 days post inoculation (DPI); the other inoculated spikelets showed only mild bleaching symptoms (Fig 6D). Fewer than 3% of spikelets were infected by the Δ*2021Famyo2-7* and Δ*Y2021AFamyo2-8* mutants at 15 DPI.

In infection assays with wheat coleoptiles, spore suspensions of *F*. *asiaticum* parental and mutant strains were injected on the cut tips of coleoptiles of 3-d-old wheat seedlings. Majority of the coleoptiles split naturally within 1 to 3 DPI, and the stems, which beginning from the inoculated apex, started to turn brown within 4 to 7 DPI. By 10 to 14 DPI, dark brown lesions were evident on the coleoptiles of the inoculated seedlings. Lesions caused by the *FaMyo2B* and *Famyo2* mutants were obviously smaller than those caused by the parent strains (Fig 6A and 6B).

Because the trichothecene toxin deoxynivalenol (DON) is a major virulence factor for *F*. *graminearum*, we assayed the expression levels of two trichothecene biosynthesis genes, *TRI5* and *TRI6*, using quantitative real-time PCR and RNA samples extracted from germ tubs grown in GYEP medium. The expression levels of *TRI5* and *TRI6* were obviously lower in Δ*Famyo2* than in the parental strains (S5A Fig).

The MAP kinases Gpmk1 and Mgv1 are required for pathogenesis in *F*. *graminearum*, and previous studies demonstrated that Gpmk1 regulates the activities of xylanolytic, extracellular endoglucanase and proteolytic enzymes [44]. To determine whether *FaMyo2B* and *Famyo2* participate in the regulation of Gpmk1 or Mgv1, we analyzed the expression levels of *FaGpmk1* and *FaMgv1* in the mutants and parental strains. The expression levels of *FaGpmk1* and *FaMgv1* were substantially down-regulated in Δ*Famyo2* (S5B Fig). These results may explain the reduced pathogenicity of the *Famyo2* deletion mutants.

### *Famyo2* is required for nucleus and septum distribution and for cell wall integrity

**Fig 7:**
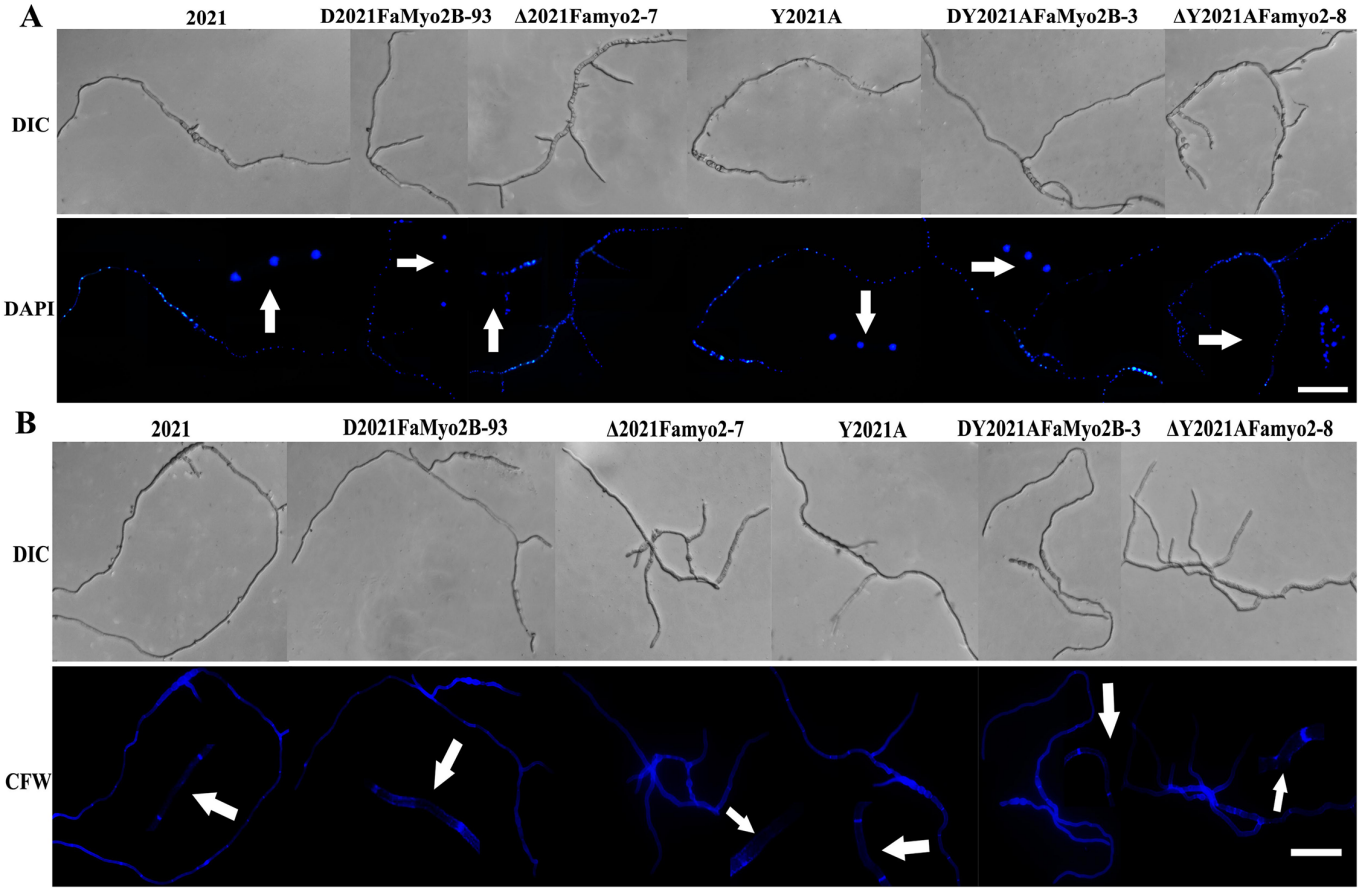
Distribution of nuclei and septa in hyphae of the wild-type strain 2021 and of the *Famyo2* mutants. (A) Nuclei were stained with DAPI after incubation for 12 h. (B) Septa were stained with CFW after incubation for 12 h. The arrow heads represent partial, enlarged results. Bar = 40 μm.

Song *et al*. (2013) [20] showed that the Class II myosin myo2 is required for septation in *F*. *graminearum*. We investigated the role of *FaMyo2B* and *Famyo2* in the distribution of nuclei and in septum development in *F*. *asiaticum* by staining hyphae of the parental strain and of the *Famyo2* and *FaMyo2B* mutants with calcofluor white (CFW) and 4’,6-diamidino-2-phenylinodole (DAPI). Analysis revealed that the nuclei were regularly distributed in the *FaMyo2B* mutant hyphae and in the parental hyphae. In contrast, they were unevenly distributed and clustered in the hyphae of the Δ*Famyo2* mutants (Fig 7A). Septum development also differed substantially between the parental and the Δ*Famyo2* mutant strains. Septa in parental hyphae were complete and uniformly distributed. In contrast, septa in Δ*Famyo2* mutant hyphae were often incomplete and irregularly distributed. Moreover, many of the Δ*Famyo2* mutant septa revealed by CFW staining could not be detected by fluorescence microscopy (Fig 7B).

Because the myosin Class II gene *Myo1* plays an important role in cell wall maintenance in yeast cells [30], we also determined the effects of the cell wall damaging agents caffeine and Congo red on the Δ*Famyo2* mutant. Compared to the parental strain, the Δ*Famyo2* mutant showed increased sensitivity to these compounds (S6 Fig). We also quantified the expression of *FaMgv1*, which is homologous to the *S*. *cerevisiae* cell wall integrity core element gene, *Slt2*. Expression levels of *FaMgv1* were slightly down-regulated in Δ*Famyo2* relative to the parental strains (S5B Fig). These results indicated that *Famyo2* is required for nucleus and septum distribution and for cell wall integrity.

## Discussion

Because the actin cytoskeleton and microtubules have important roles in key cellular events, they are attractive targets for drug design. The actin cytoskeleton is composed of polymers of actin (microfilaments) together with actin-binding and actin-associated proteins, such as cytoskeletal motor myosins [45, 46]. Myosins are important components of the eukaryotic cytoskeleton because they provide motility for many kinds of cargo. We previously reported that the actin-bundling protein fimbrin was important in regulating the level of phenamacril resistance in *F*. *graminearum* [17]. We then demonstrated that mutations in the class I myosin gene *myosin5* is responsible for resistance to phenamacril [18]. In this paper, we demonstrated that FaMyo2B acts jointly with the myosin passenger protein FaSmy1 to affect phenamacril resistance in *F*. *asiaticum*.

Class V myosins have a myosin head domain and a tail domain with IQ and globular DIL domains. In budding yeast, the class V myosin Myo2p and the myosin passenger protein Smy1p have an intimate relationship, as determined by co-localization and physical interaction [21, 33, 34]. Furthermore, overexpression of Smy1p not only complements the localization of the mutated Myo2p but also enhances the localization of the wild-type Myo2p [34]. In this study, a yeast two hybrid assay revealed that FaSmy1 interacts with the FaMyo2B tail in *F*. *asiaticum*. To our surprise, disruption of either *FaMyo2B* or *FaSmy1* in the phenamacril-resistant strain Y2021A significantly reduced phenamacril EC_50_ values. And when we complemented the *FaMyo2B* disruption mutants with full-length *FaMyo2B* gene, the complementations partially restored the resistance of the mutants, which indicated that the FaMyo2B protein played an important role in the resistance to phenamacril in *F*.*asiaticum*. In addition, disruption of *FaMyo2B* in *F*. *asiaticum* also significantly down-regulated the expression of *FaSmy1* in the phenamacril-resistant strain Y2021A and in the phenamacril-sensitive strain 2021, which is consistent with previous studies with *S*. *cerevisiae* indicating that Smy1 accumulation in the bud tip needs Myo2 and that Smy1 is trafficked by Myo2 on actin cables [21, 31, 34, 47]. In the current study, treatment with 1 μg/ml phenamacril for 6 h caused the D*Y2021AFaMyo2B-3* disruption mutant to significantly up-regulate *FaMyo5*, *FaFim*, and *FaSmy1* genes relative to the sensitive strain 2021. These results help us better understand the resistance mechanism.

In *S*. *cerevisiae*, Myo2 encodes an essential gene. The failure to obtain an *FaMyo2B* null mutant for *F*. *asiaticum* strongly indicated that deletion of *FaMyo2B* has a lethal effect in this fungus. However, we selected several *FaMyo2B* heterokaryotic disruption mutants for further study. We found that the *FaMyo2B* disruption mutant and the *Famyo2* deletion mutant exhibited defective hyphal branching, reduced asexual reproduction, and no sexual reproduction. As we know, *Fusarium* species are mononuclear fungus, but the heterokaryotic mycelial of *FaMyo2B* disruption mutants have two types of genes, one is deletion type and another is wild-type. Maybe the lethal impact lead to this phenomenon in this fungus. Interestingly, the *FaMyo2B* heterokaryotic disruption mutants also caused the changes of phenotype like deletion mutants, which indicated that *FaMyo2B* has a direct or indirect impact on asexual or sexual reproduction.

Yeast cells carry on cell divisions by separating the daughter cells, while filamentous fungi form compartments divided by septa [48]. We discovered that septal formation in the hyphae of the *Famyo2* mutants was seriously disrupted, which resulted in unevenly distributed and clustered nuclei. The *Famyo2* deletion mutant also exhibited abnormal conidial development and enhanced sensitivity to cell wall damaging agents, which revealed that *Famyo2* is essential for cell wall integrity. In addition, the production of perithecia was abolished in the *Famyo2* mutants on carrot agar plates, indicating a major role of the Famyo2 protein in the development of *F*. *asiaticum*. Although the *FaMyo2B* mutant produced normal perithecia, the ascospores within those perithecia failed to germinate. Ascospores released by *F*. *graminearum* perithecia are required for the primary infection of wheat spikes during wheat flowering [49]. The defective sexual reproduction of the *Famyo2* and *FaMyo2B* mutants suggests that *Famyo2* and *FaMyo2B* or the proteins they encode could be the targets for new drugs that control FHB.

In the current study, the *Famyo2* and *FaMyo2B* mutants were less pathogenic than the wild types on flowering wheat heads and wheat coleoptiles. In addition to having reduced septation and enhanced sensitivity to cell wall damaging agents, the Δ*Famyo2* mutant had reduced expression levels of *FaGpmk1*, resulting in reduced penetration of host tissue. Expression levels of the trichothecene biosynthesis genes *TRI5* and *TRI6* were also reduced in the Δ*Famyo2* mutant. These genes are responsible for the synthesis of DON, which is required for the spread of *F*. *graminearum* in rachis tissue [50]. The reduced expression levels of *TRI5* and *TRI6* are consistent with the failure of the Δ*Famyo2* mutant to spread *in planta*. Although mycelial growth was only slightly reduced and *TRI5* and *TRI6* expression were normal in the *FaMyo2B* disruption mutant, its virulence was reduced on flowering wheat heads and wheat coleoptiles. We suspect that the disruption of *FaMyo2B* affects the transport of cargo along the actin cable, which results in downstream defects that reduce pathogenicity.

**Fig 8:**
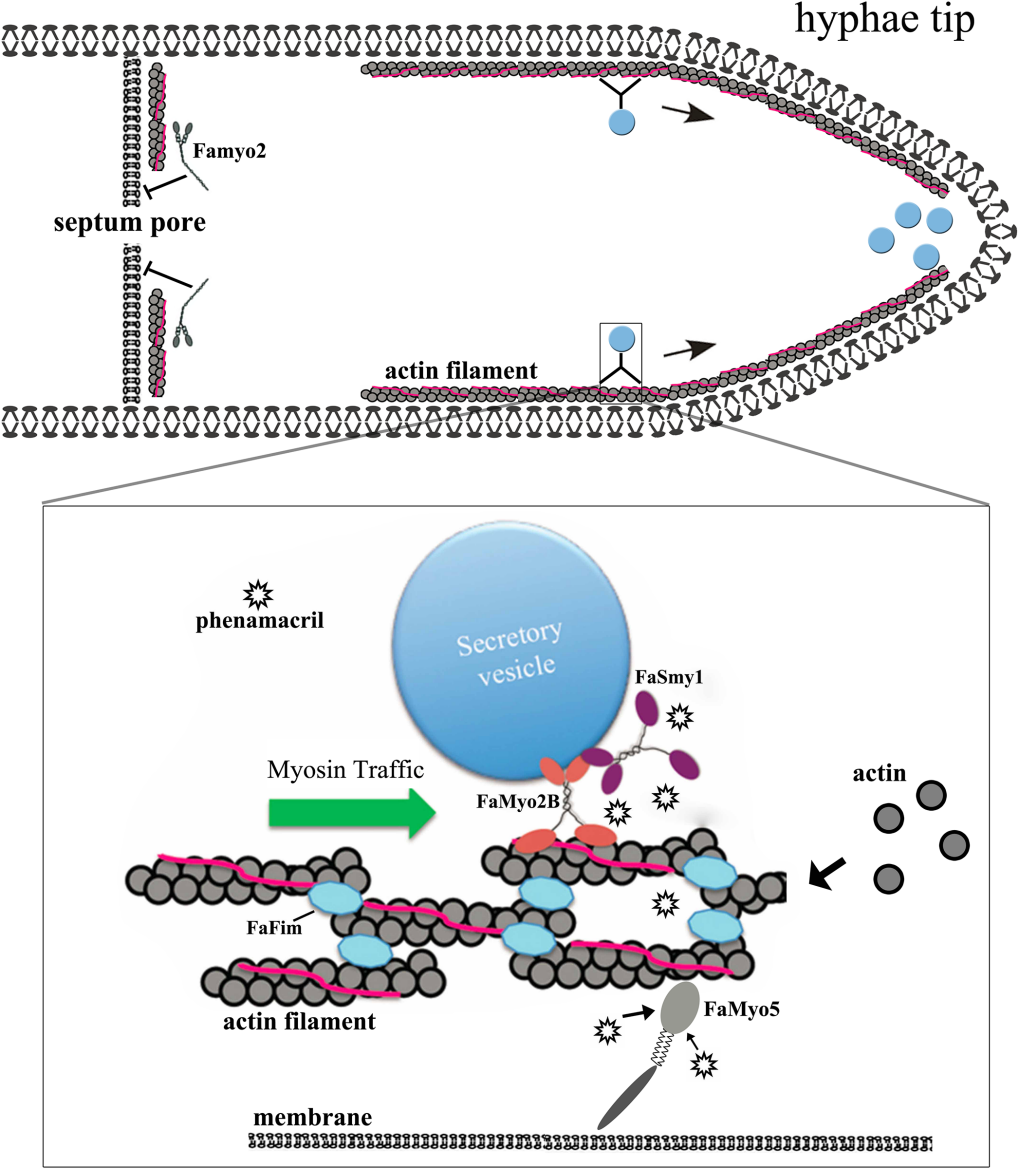
A hyphae tip cell uses bilayer membrane. Myosin transports secretory vesicles to hyphae tips along the actin cables. Famyo2 controls septum development. Box, the actin filaments in cables contained actin bundling proteins (FaFim) and tropomyosin (pink), which maintain filament stability and organization. Myosin-V (FaMyo2B, red) transports FaSmy1 (purple) and secretory vesicles on actin cables to the hyphae tips. FaMyo5 (grey) trigger actin polymerization and travel toward the hyphae tips. By binding to FaMyo5 in phenamacril-sensitive strains (the fungicide is indicated by asterisks), the fungicide disrupts transport along the actin cables. In phenamacril-resistant strains, the binding of phenamacril to FaMyo5 is reduced, and the disruption in transport is therefore reduced.

Identifying drug targets and drug resistance mechanisms is difficult because many proteins and complex and systemic pathways are involved. Finally, we present a model for phenamacril resistance in *F*. *asiaticum* (Fig 8). In the absence of phenamacril, the actin-bundling protein FaFim stables the actin cable; FaMyo5 always trigger Arp2/3 complex-dependent actin polymerization and travel toward the hyphae tips along the actin cable; FaMyo2B and FaSmy1 (the myosin passenger protein) transport secretory vesicles along the actin cable; and Famyo2 maintains cell wall integrity and controls septum development. When a phenamacril-sensitive strain is treated with the fungicide, phenamacril binds to FaMyo5 or inhibits ATPase activity of FaMyo5 motor domain and thereby reduces actin polymerization and the transport of secretory vesicles along the actin cable; this can greatly disrupt cell functions and hyphae growth. Resistance to phenamacril results from mutations in myosin-5, which apparently reduces the binding of the fungicide to FaMyo5. While mutations in FaMyo5 result in phenamacril resistance, the disruption of *FaMyo2B* and deletion of *FaSmy1* significantly reduced phenamacril resistance for the phenamacril-resistant strain (Y2021A), which resulted from the disrupted transport of secretory vesicles. In summary, our results revealed a logical explanation for phenamacril resistance and we needed more experiments to prove.

## Supporting Information

S1 Fig Schematic representation of *Fusarium asiaticum* FaMyo2B and Famyo2. The conserved motor domain, myosin tail (TH1), and src homology domain 3 (SH3) are highlighted.
(DOC)

S2 Fig The alignment of the amino acid sequences of the FaMyo2B motor domain with those from *Neurospora crassa*, *Botrytis cinerea*, and *Saccharomyces cerevisiae*. (DOC)

S3 Fig The alignment of the amino acid sequences of the Famyo2 motor domain with those from *Neurospora crassa*, *Botrytis cinerea*, and *Saccharomyces cerevisiae*. (DOC)

S4 Fig Generation and identification of *FaMyo2B* gene disruption mutants and *Famyo2* gene deletion mutants. (A) Gene replacement strategy for *FaMyo2B* and *Famyo2*. The gene replacement cassette HPH-HSV-tk contains the hygromycin resistance gene and the herpes simplex virus thymidine kinase gene. Primer binding sites are indicated by arrows (see S1 Table for the primer sequences). (B) PCR analysis for identification of *FaMyo2B* and *Famyo2* mutants. (C) Southern blot hybridization analysis of *FaMyo2B* mutants using the 576-bp upstream DNA fragment of *FaMyo2B* as a probe and genomic DNA are digested with Hind III. (D) Southern blot hybridization analysis of *Famyo2* mutants using the 661-bp downstream DNA fragment of *Famyo2* as probe and genomic DNA are digested with Cla I.
(DOC)

S5 Fig Expression level of genes that are essential for virulence. (A) Expression level of *TIR5* and *TRI6* in mutants relative to expression in strain 2021. (B) Expression level of *FaMgv1* and *FaGpmk1* in mutants relative to expression in strain 2021. Values are the means ± SE of three repeated experiments.
(DOC)

S6 Fig Effects of Famyo2 on the sensitivity of *Fusarium asiaticum* strains to cell wall-damaging agents (Congo red and caffeine). Values are the means ± SE of three repeated experiments.
(DOC)

S1 Table Oligonucleotide primers used in this study. (DOC)
