# Supplementary material for: Myosins FaMyo2B and Famyo2 Affect Asexual and Sexual Development, Reduces Pathogenicity, and FaMyo2B Acts Jointly with the Myosin Passenger Protein FaSmy1 to Affect Resistance to Phenamacril in Fusarium asiaticum
Source: PLoS One. 2016 Apr 21;11(4):e0154058. doi: 10.1371/journal.pone.0154058 (PMC4839718; doi:10.1371/journal.pone.0154058)

**S1 Fig. Schematic representation of *Fusarium asiaticum* FaMyo2B and Famyo2.** The conserved motor domain, myosin tail (TH1), and src homology domain 3 (SH3) are highlighted.


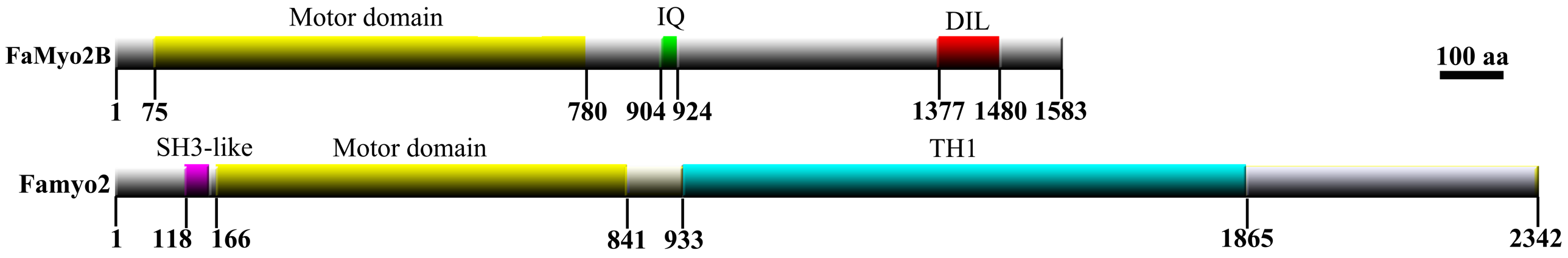

Supplement: S1 Fig — The conserved motor domain, myosin tail (TH1), and src homology domain 3 (SH3) are highlighted. (DOC) [file pone.0154058.s001.doc]
