# Supplementary material for: Myosins FaMyo2B and Famyo2 Affect Asexual and Sexual Development, Reduces Pathogenicity, and FaMyo2B Acts Jointly with the Myosin Passenger Protein FaSmy1 to Affect Resistance to Phenamacril in Fusarium asiaticum
Source: PLoS One. 2016 Apr 21;11(4):e0154058. doi: 10.1371/journal.pone.0154058 (PMC4839718; doi:10.1371/journal.pone.0154058)

**S2 Fig. The alignment of the amino acid sequences of the FaMyo2B motor domain with those from *Neurospora crassa*, *Botrytis cinerea*, and *Saccharomyces cerevisiae*.**


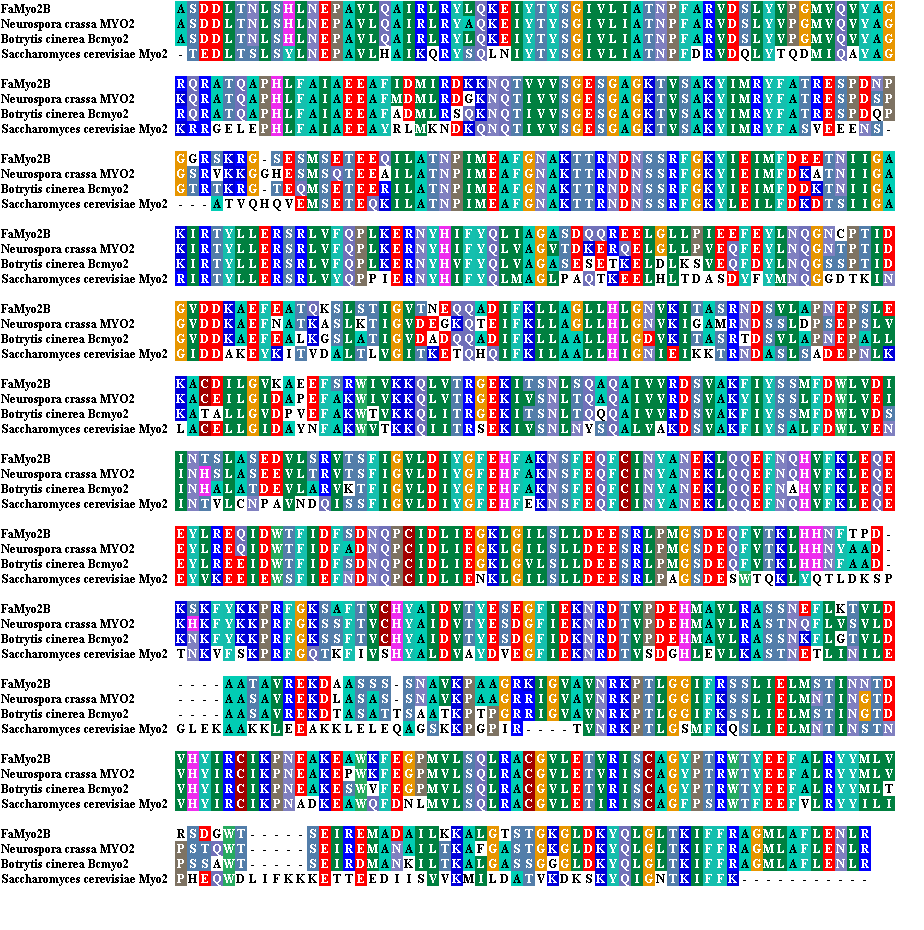

Supplement: S2 Fig — (DOC) [file pone.0154058.s002.doc]
