# Supplementary material for: Myosins FaMyo2B and Famyo2 Affect Asexual and Sexual Development, Reduces Pathogenicity, and FaMyo2B Acts Jointly with the Myosin Passenger Protein FaSmy1 to Affect Resistance to Phenamacril in Fusarium asiaticum
Source: PLoS One. 2016 Apr 21;11(4):e0154058. doi: 10.1371/journal.pone.0154058 (PMC4839718; doi:10.1371/journal.pone.0154058)

**S4 Fig. Generation and identification of *FaMyo2B* gene disruption mutants and *Famyo2* gene deletion mutants.** (A) Gene replacement strategy for *FaMyo2B* and *Famyo2*. The gene replacement cassette HPH-HSV-tk contains the hygromycin resistance gene and the herpes simplex virus thymidine kinase gene. Primer binding sites are indicated by arrows (see Table S1 for the primer sequences). (B) PCR analysis for identification of *FaMyo2B* and *Famyo2* mutants. (C) Southern blot hybridization analysis of *FaMyo2B* mutants using the 576-bp upstream DNA fragment of *FaMyo2B* as a probe and genomic DNA are digested with HindⅢ. (D) Southern blot hybridization analysis of *Famyo2* mutants using the 661-bp downstream DNA fragment of *Famyo2* as probe and genomic DNA are digested with Cla I.


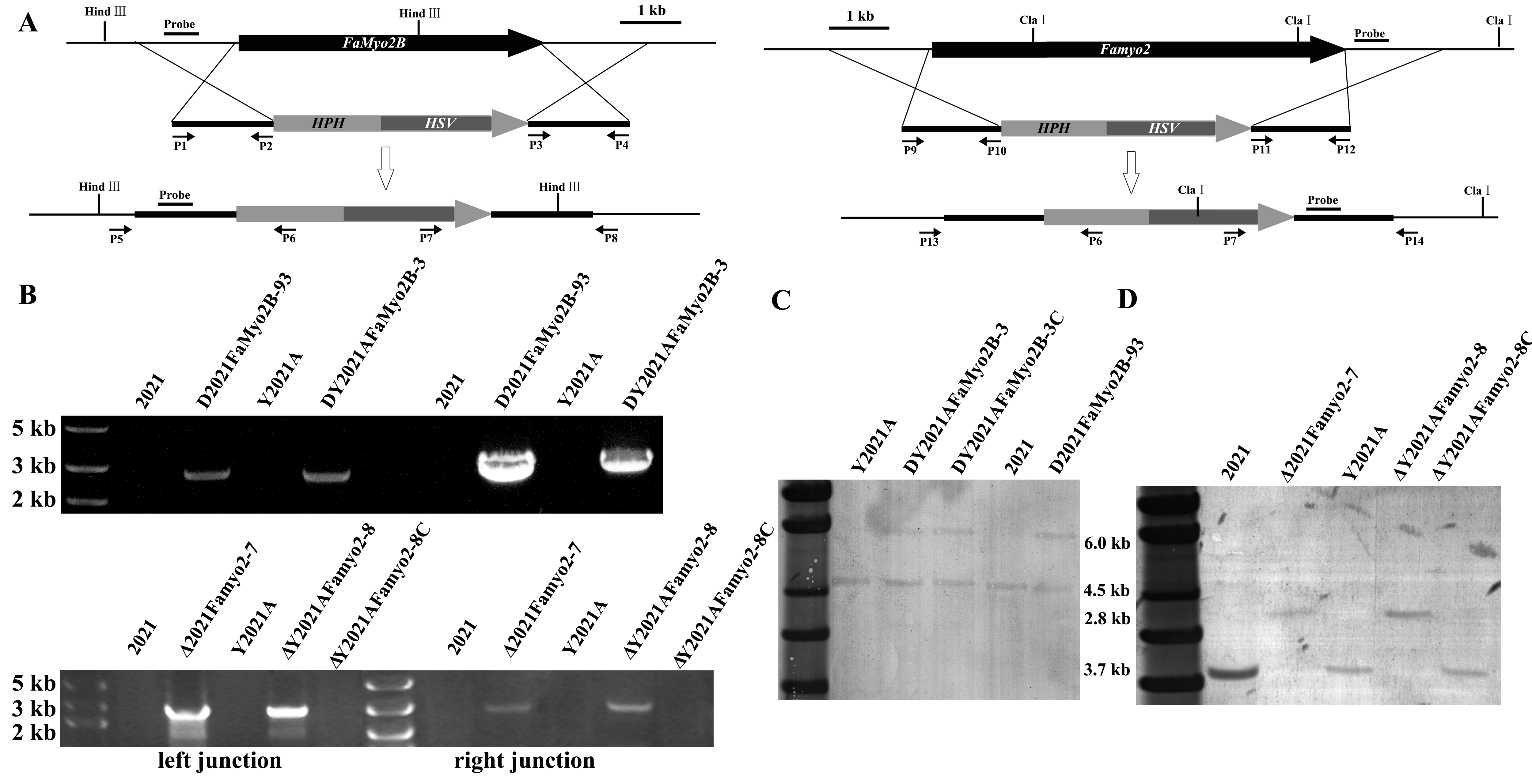

Supplement: S4 Fig — (A) Gene replacement strategy for FaMyo2B and Famyo2. The gene replacement cassette HPH-HSV-tk contains the hygromycin resistance gene and the herpes simplex virus thymidine kinase gene. Primer binding sites are indicated by arrows (see S1 Table for the primer sequences). (B) PCR analysis for identification of FaMyo2B and Famyo2 mutants. (C) Southern blot hybridization analysis of FaMyo2B mutants using the 576-bp upstream DNA fragment of FaMyo2B as a probe and genomic DNA are digested with Hind III. (D) Southern blot hybridization analysis of Famyo2 mutants using the 661-bp downstream DNA fragment of Famyo2 as probe and genomic DNA are digested with Cla I. (DOC) [file pone.0154058.s004.doc]
