# Supplementary material for: Myosins FaMyo2B and Famyo2 Affect Asexual and Sexual Development, Reduces Pathogenicity, and FaMyo2B Acts Jointly with the Myosin Passenger Protein FaSmy1 to Affect Resistance to Phenamacril in Fusarium asiaticum
Source: PLoS One. 2016 Apr 21;11(4):e0154058. doi: 10.1371/journal.pone.0154058 (PMC4839718; doi:10.1371/journal.pone.0154058)

**S5 Fig. Expression level of genes that are essential for virulence.** (A) Expression level of *TIR5* and *TRI6* in mutants relative to expression in strain 2021. (B) Expression level of *FaMgv1* and *FaGpmk1* in mutants relative to expression in strain 2021. Values are the means ± SE of three repeated experiments.


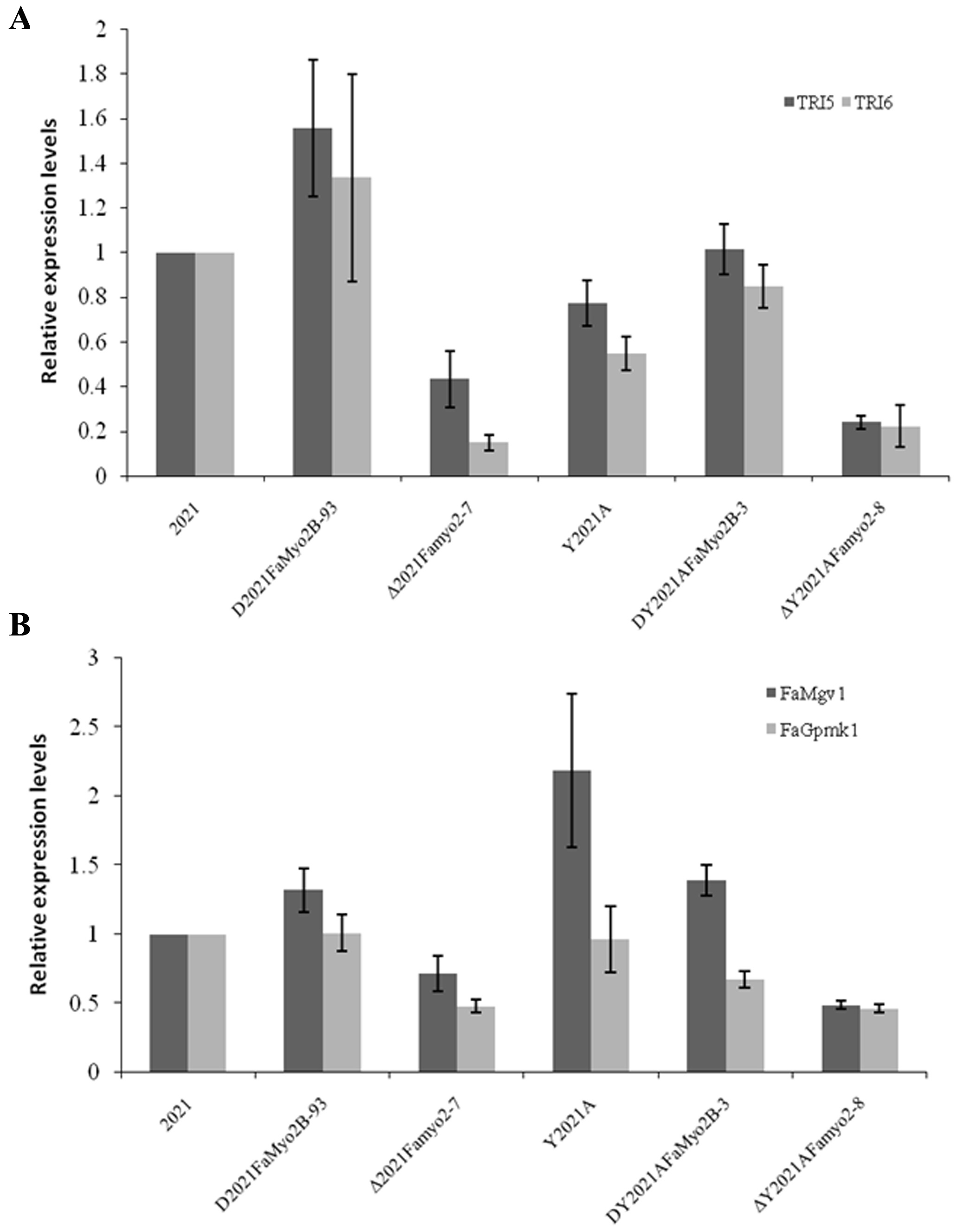

Supplement: S5 Fig — (A) Expression level of TIR5 and TRI6 in mutants relative to expression in strain 2021. (B) Expression level of FaMgv1 and FaGpmk1 in mutants relative to expression in strain 2021. Values are the means ± SE of three repeated experiments. (DOC) [file pone.0154058.s005.doc]
