# Supplementary material for: Myosins FaMyo2B and Famyo2 Affect Asexual and Sexual Development, Reduces Pathogenicity, and FaMyo2B Acts Jointly with the Myosin Passenger Protein FaSmy1 to Affect Resistance to Phenamacril in Fusarium asiaticum
Source: PLoS One. 2016 Apr 21;11(4):e0154058. doi: 10.1371/journal.pone.0154058 (PMC4839718; doi:10.1371/journal.pone.0154058)

**S6 Fig. Effects of Famyo2 on the sensitivity of *Fusarium asiaticum* strains to cell wall-damaging agents (Congo red and caffeine).** Values are the means + SE of three repeated experiments.


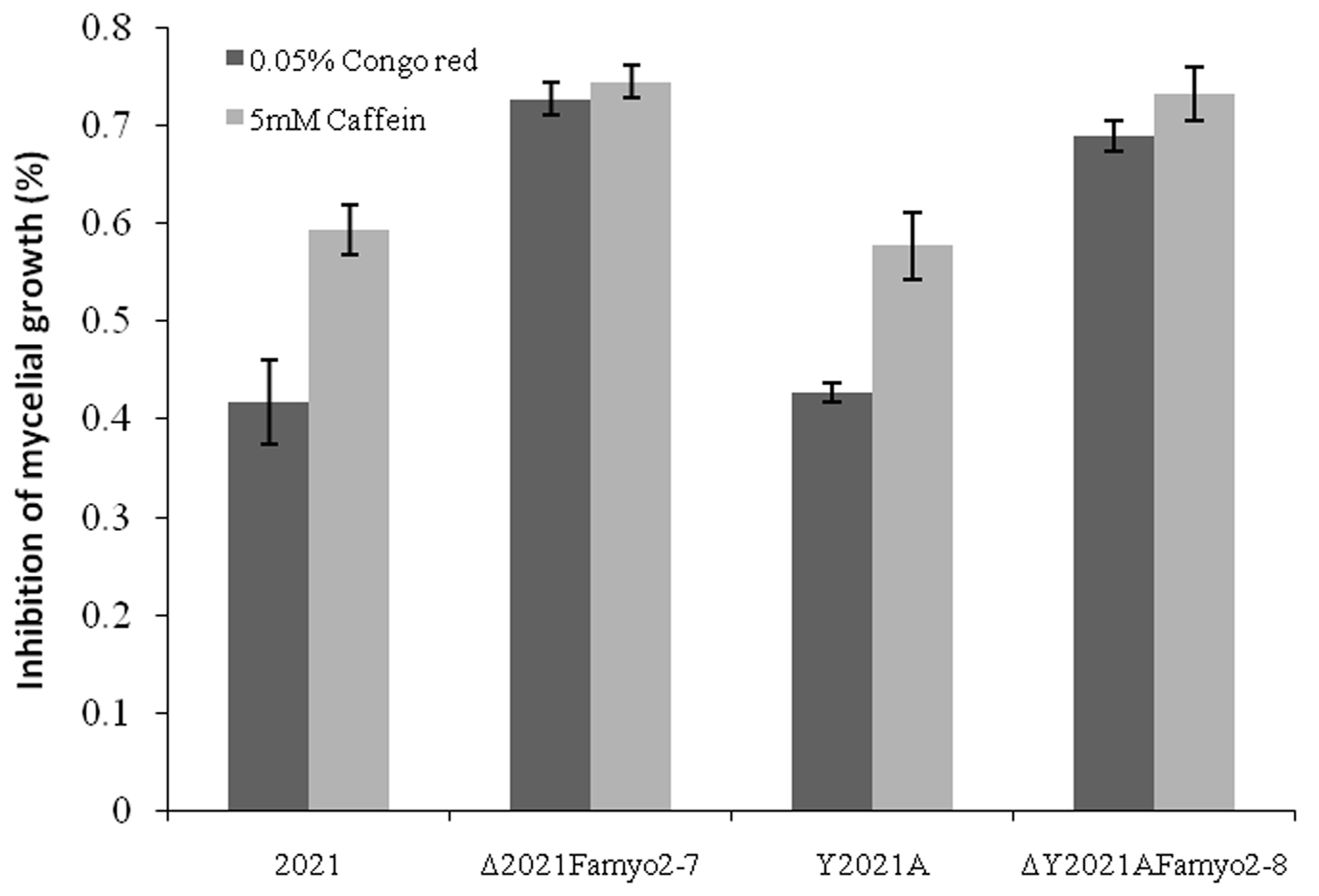

Supplement: S6 Fig — Values are the means ± SE of three repeated experiments. (DOC) [file pone.0154058.s006.doc]
