# Supplementary material for: Myosins FaMyo2B and Famyo2 Affect Asexual and Sexual Development, Reduces Pathogenicity, and FaMyo2B Acts Jointly with the Myosin Passenger Protein FaSmy1 to Affect Resistance to Phenamacril in Fusarium asiaticum
Source: PLoS One. 2016 Apr 21;11(4):e0154058. doi: 10.1371/journal.pone.0154058 (PMC4839718; doi:10.1371/journal.pone.0154058)
Supplement: S1 Table — (DOC) [file pone.0154058.s007.doc]

**S1 Table. Oligonucleotide primers used in this study.**

| Primer code | Primer | Sequence (5’-3’)**^a^** | Relevant characteristics |
| --- | --- | --- | --- |
| 1 | 07469F | AGCGTGTCGGTTGTTTGC | PCR primers for amplification of the full *FaMyo2B* fragment |
| 2 | 07469R | ATGCCTTCCAGATACAACG |
| 3 | 08719F | TTTCCCGTTGCAGTTGCA | PCR primers for amplification of the full *Famyo2* fragment |
| 4 | 08719R | CATCTGCGAAGCGAACAC |
| 5 | P1 | CGTCTACTTGGCGTGCTA | PCR primers for amplification of the upstream *FaMyo2B* fragment |
| 6 | P2 | TTCAATATCATCTTCTGTGATGGCGGTATTACCGAG |
| 7 | P3 | GACAATACCGGAAGGAACAGAGATAATGAAGCTGGT | PCR primers for amplification of the downstream *FaMyo2B* fragment |
| 8 | P4 | TGTGACCTCCTCAAGCGT |
| 9 | P5 | ATGCTGTATCTGGGAGGC | A pair of PCR primers for identification of D*FaMyo2B* integration at the left junction. |
| 10 | P6 | GGTGTCGTCCATCACAGTTTGC |
| 11 | P7 | CCGCCTTGACCAGGGTGAGAT | A pair of PCR primers for identification of D*FaMyo2B* integration at the left junction. |
| 12 | P8 | GACCTTTGAGGGCATTGT |
| 13 | P9 | CTCCATCTTTGGTTCTGCTC | PCR primers for amplification of the upstream *Famyo2* fragment |
| 14 | P10 | TTCAATATCATCTTCTGTCGCGAAGGGCCTGCATGA |
| 15 | P11 | GACAATACCGGAAGGAACGGCGGCGTGTATGAGAAG | PCR primers for amplification of the downstream *Famyo2* fragment |
| 16 | P12 | CGTGGGAAGAACGAAGGTA |
| 17 | P13 | GGCAATCTCGGTTGTTCC | A pair of PCR primers for identification of Δ*Famyo2* integration at the left junction. |
| 18 | P6 | GGTGTCGTCCATCACAGTTTGC |
| 19 | P7 | CCGCCTTGACCAGGGTGAGAT | A pair of PCR primers for identification of Δ*Famyo2* integration at the left junction. |
| 20 | P14 | ATGGGAGGTTCGGAGGTC |
| 21 | P15 | tctagaATGCTGTATCTGGGAGGC | PCR primers for amplification of the full-length *FaMyo2B* fragment |
| 22 | P16 | cctgcaggATGCCTTCCAGATACAACG |
| 23 | 07469ProbeF | CGATTTAGATTTCGGAGGTG | PCR primers to amplify the 576-bp upstream DNA fragment of *FaMyo2B* fragment used as a probe for Southern blot |
| 24 | 07469ProbeR | CAAAAGTTATGGATGCGGTG |
| 25 | 08719ProbeF | CTGAGTTCGCTCCCTTAGTT | PCR primers to amplify the 661-bp downstream DNA fragment of *Famyo2* fragment used as a probe for Southern blot |
| 26 | 08719ProbeR | TGTGAGATGGTATGCGTTTA |
| 27 | Tri5-rtF | GAGTGTTTCATGCATGGCTACGTC | Quantitative real-time PCR primers for analysis of *TRI*5 expression |
| 28 | Tri5-rtR | CTGAGCCTCCTTCACATCGTCC |
| 29 | Tri6-rtF | TATCGAAAATTATATAACCACATC | Quantitative real-time PCR primers for analysis of *TRI*6 expression |
| 30 | Tri6-rtR | CTGAGGGCATTCTGAGTAGCGACA |
| 31 | Mgv1-rtF | TTCTTCACATTCTCGGAACCC | Quantitative real-time PCR primers for analysis of *FaMgv1* expression |
| 32 | Mgv1-rtR | CATCCAAAATCATACCACGCA |
| 33 | Gpmk1-rtF | TGTCGTCTGTTCCGCCATTC | Quantitative real-time PCR primers for analysis of *FaGpmk1* expression |
| 34 | Gpmk1-rtR | TGTCGTCTGTTCCGCCATTC |
| 35 | FaMyo5rtF | TCACAAGTCCCAGCCGATTA | Quantitative real-time PCR primers for analysis of *FaMyo5* expression |
| 36 | FaMyo5rtR | GGTTGCCTTGAATGCGAGA |
| 37 | FaMyo2BrtF | GCAGTCCCTCGGTTCTATGA | Quantitative real-time PCR primers for analysis of *FaMyo2B* expression |
| 38 | FaMyo2BrtR | CTGGTTGGCTTCCGTCTGT |
| 39 | Famyo2rtF | GCCTTGGACAGGGTTTCATT | Quantitative real-time PCR primers for analysis of *Famyo2* expression |
| 40 | Famyo2rtR | TCCTCCTCAGATTCGGCAC |
| 41 | FimrtF | CCTCCAAGAAGCGGACAAGC | Quantitative real-time PCR primers for analysis of FaFim expression |
| 42 | FimrtR | GAGCCACAAAGTGAAAACCC |
| 43 | msy1rtF | ACACTGCGATTTGGTATGCG | Quantitative real-time PCR primers for analysis of FaMsy1 expression |
| 44 | msy1rtR | CCCTCTCGTCCTTGTCCAGT |
| 45 | GAPDH-F | CTTACTGCCTCCACCAACTG | Quantitative real-time PCR primers for analysis of glyceraldehyde-3-phosphate dehydrogenase expression |
| 46 | GAPDH-R | TGACGTTGGAAGGAGCGAAG |
| 47 | Smy1BKF | GGAATTCCATATGATGTCGTCCGCAAATAGT | Primer for yeast two-hybrid analysis of pGBKT7-FaSmy1 |
| 48 | Smy1BKR | CGCGGATCCCTATGACTTGGCGAAGAA |
| 49 | Myo2BADF | CCGGAATTCACGACCAGGCTCAACGAC | Primer for yeast two-hybrid analysis of pGADT7-FaMyo2B |
| 50 | Myo2BADR | CGCGGATCCTTAATGACCGTTCTCGTATTC |
| 51 | myo2ADF | CCGGAATTCGCCCTCATTACTCAGATC | Primer for yeast two-hybrid analysis of pGADT7-Famyo2 |
| 52 | myo2ADR | CGCGGATCCTTAAGGGATGTGGACCTCTGG |

**^a^** Lowercase letters indicate restriction enzyme sites in the primers.
